# 1395. Epidemiology of Non-Tuberculous Mycobacteria Infection Among Immunocompromised Children: A Single Center Experience

**DOI:** 10.1093/ofid/ofab466.1587

**Published:** 2021-12-04

**Authors:** Ye jin Kang, Paul K Sue, Laura Filkins, Giselle S Chery

**Affiliations:** 1 UT Southwestern, Dallas, Texas; 2 UT Southwestern Medical Center, Dallas, TX; 3 University of Texas Southwestern/Children’s Health, Dallas, Texas

## Abstract

**Background:**

Nontuberculous mycobacteria (NTM) infection is associated with high rates of morbidity and mortality among immunocompromised adults. However, sparse data exists regarding clinical outcomes among immunocompromised (IC) children with NTM infection. We sought to characterize clinical features and outcomes among IC children at our institution with microbiologically confirmed NTM disease.

**Methods:**

Retrospective review of cases of microbiologically confirmed NTM infection among IC children between January 2017 and December 2020. Children (≤21y.o) with microbiologically confirmed NTM disease and known primary or secondary immunodeficiency diagnosed between January 1, 2017 and December 20, 2020 were included in the study. All subjects with a positive NTM microbiologic stain or culture but no subsequent treatment for NTM infection were excluded. Demographic and clinical characteristics were assessed and risk factors for mortality were evaluated.

**Results:**

Of 147 mycobacterial cultures sent during the study period, 72 subjects had a positive microbiologically confirmed NTM species, with 10 subjects meeting all inclusion and no exclusion criteria. Median age was 16 years old, with 40 percent being female and 50 percent of Hispanic ethnicity. NTM disease was distributed among patients with primary immunodeficiency (30%), solid organ transplantation (20%), hematopoietic stem cell transplant (20%), rheumatologic disease on immunosuppressive therapy (10%), and hematologic malignancy (10%). Bacteremia was common, with blood cultures positive in 70% of cases, and *M. abscessus (50%) and M. avium complex (30%*) the most frequently implicated pathogens. Hospital acquired infection was common (60%). 2 year mortality following invasive NTM infection was high at 40%.

**Conclusion:**

While rare, NTM infections are associated with significant morbidity and mortality among immunocompromised children. Additional investigations are needed to assess for risk factors associated with NTM and severe NTM disease.

**Disclosures:**

**Laura Filkins, PhD**, **Avsana Labs** (Board Member, Scientific advisory board member)**Biofire Diagnostics** (Grant/Research Support)

